# Microbial production of toluene in oxygen minimum zone waters in the Humboldt Current System off Chile

**DOI:** 10.1038/s41598-022-14103-2

**Published:** 2022-06-23

**Authors:** Benjamín M. Srain, Silvio Pantoja-Gutiérrez

**Affiliations:** 1 Departamento de Oceanografía and Centro de Investigación Oceanográfica COPAS Sur-Austral, Universidad de Concepción, Concepción, Chile; 2 Centro de Investigación Oceanográfica COPAS Coastal, Universidad de Concepción, Concepción, Chile; 3 Present Address: Programa de Estudios Ecosistémicos del Golfo de Arauco (PREGA), Universidad de Concepción, Concepción, Chile

**Keywords:** Biogeochemistry, Carbon cycle, Element cycles

## Abstract

Expansion of oxygen minimum zones in the world's oceans is likely to enhance the production of anaerobic metabolites by marine microorganisms. Here we show that toluene is present throughout the year in shelf waters of the upwelling ecosystem off Concepción (36° S), Chile, and it is a product of microbial anaerobic metabolism. The intra-annual variability in toluene concentrations is consistent with seasonal variability in the strengths of suboxic equatorial and oxygenated subantarctic water masses. Laboratory incubations of oxygen minimum zone water showed microbial production of toluene in the absence of O_2_. Toluene concentrations were elevated (up to 96 nM) in deeper O_2_-depleted waters and followed a seasonal pattern in oceanographic conditions. There is evidence to hypothesize that microbial production of toluene could be a homeostatic biochemical mechanism to thrive in the more acidic oxygen minimum zone waters. On the other hand, evidence indicates that microbial anaerobic degradation of toluene may be a source of NO_2_^−^ by partial denitrification, as shown for aquifer sediments. Since toluene production was not detected in incubations under aerobic conditions, we hypothesize that oxygen minimum zone waters export toluene to surrounding oxygenated waters. Expansion of hypoxia in the ocean will certainly enhance the production and export of anaerobic metabolites by marine microorganisms.

## Introduction

Across the world’s oceans, anaerobic metabolism presently occurs in marine sediments, anoxic basins, and areas known as oxygen minimum zones (OMZ). These OMZ regions are open ocean waters^1^, occupying approximately 1% of the global ocean volume, but with a disproportionate influence on biogeochemical cycles through the removal of nitrogen and greenhouse gases from the ocean to the atmosphere^2^. Documented expansion of OMZ regions across the world's oceans^3^ will expand anaerobic microbial metabolism in the water column and the associated production of reduced metabolites^4–8^ readily available for further microbial degradation.

Toluene is an aromatic hydrocarbon that occurs as a minor component of gasoline and is also produced by bacteria^9,10^ through anaerobic decarboxylation of phenylacetate derived from the catabolism of the amino acid phenylalanine^10,11^. Biological production of toluene has been reported in anaerobic bacterial isolates^12^, anaerobic sewage sludge^13,14^, anoxic freshwater sediments^15,16^, and the anoxic hypolimnion of stratified lakes^17^. Marine phytoplankton is also a potential source of toluene, as shown in monocultures of coccolithophores and diatoms under oxidative stress. They could be a potential source of benzenoids to the atmosphere^18^.

In oceanic OMZ waters, biogenic production of toluene is promoted both by low-O_2_ conditions and by the high availability of proteinaceous organic matter (phenylalanine). As a permanent feature of the modern ocean, these OMZ waters are maintained by elevated fluxes of plankton-derived material from the photic zone, mainly in the form of protein carbon that in turn promotes oxygen depletion^19^. The biological production of toluene from phenylalanine has been shown to involve transamination, decarboxylation, and oxidation to form phenylacetate^10^, which is the substrate for a glycyl radical decarboxylase enzyme that catalyzes phenylacetate decarboxylation to toluene^9,10,20^, a process that is irreversibly inactivated in the presence of O_2_^10,19,21^. Genomic evidence indicates that glycyl radical enzymes are widespread amongst facultative and obligate anaerobic microorganisms^9,22–25^.

Toluene is produced in bulk for industrial applications and consumer products and is found in concentrations of 2–4 mg/L in sewage sludge^13,23^. Toluene is a molecule of interest as a health hazard to humans^24^, and can account for a significant fraction of volatiles in the environment (compounds of high vapor pressure and low water solubility, with boiling points ranging between 69 and 317 °C)^25^. Toluene has been quantified in Narraganset Bay waters on the east coast of the US (0.2–60 nM)^26^, in the vicinity of a production platform in the Northwest Gulf of Mexico (21 nM)^27^, and Kuwait Bay (0.4–37 nM)^28^. These concentrations of toluene were attributed to the result of human activity and are higher than those reported in surface waters of the Caribbean Sea (0.5–4 nM)^29^, Resurrection Bay in the Gulf of Alaska (1–2 nM)^30^, Croatian coastal marine waters (2–9 nM)^31^, surface coastal waters of the Gulf of Mexico (0.05–4 nM)^32^, and the Chemotaxis Dock in Woods Hole (0.1 nM)^29,33^.

In the present study, we examined the microbial production of toluene in the water column of the upwelling ecosystem off Concepción in central Chile, both through laboratory incubations of OMZ waters and through monthly field oceanographic campaigns over an annual cycle. We hypothesized that toluene is a secondary metabolic product of microbial degradation of proteinaceous derived precursors under anaerobic conditions in the water column of the OMZ.

## Results

### Experimental microbial synthesis of toluene and decay of phenylalanine

Toluene was detected both in anoxic incubations with phenylalanine and in the field by GC–MS, as evidenced by m/z fragments 65 ([M − H^+^]–C_2_H_2_), the base peak tropylium cation 91 [M − H^+^] and the molecular cation 92 [M^+^] (Fig. 2A,D). Likewise, deuterated toluene (C_6_D_5_–CH_3_, toluene-*d*_*5*_) was detected in anoxic incubation bottles amended with deuterated phenylalanine. Mass spectra showed the diagnostics m/z fragments 68 ([M − H^+^]–C_2_D_2_), tropylium cation 96 [M − H^+^], and molecular ion 97 [M^+^] (Fig. 2A,E). In the 5-day anoxic incubations with phenylalanine (Fig. 3), toluene net production peaked on the third day, reaching 131 ± 3 µM toluene and 102 ± 2 µM toluene-*d*_*5*_ under treatment conditions for denitrification (Fig. 3A); 26 ± 1 µM toluene and 35 ± 8 µM toluene-*d*_*5*_ under conditions for sulfate reduction (Fig. 3B); and 82 ± 7 µM toluene and 94 ± 9 µM toluene-*d*_*5*_ in the absence of added external electron acceptors (Fig. 3C). Toluene and toluene-*d*_*5*_ were below the limit of detection and quantification in oxic incubations (Fig. 3D) and in control incubations (Supplementary Fig. 1A). Net maximum production of toluene and toluene-*d*_*5*_ in the presence of SO_4_^2−^ was ca. 3 times lower than in incubations with NO_3_^−^ and incubations without electron acceptors (Wilcoxon test p < 0.05).

Most (95%) phenylalanine and phenyl-*d*_*5*_-alanine initially present decayed within 5 days of incubation under all conditions (Fig. 3), without significant differences within and between experimental treatments (Wilcoxon test, p > 0.05). A significantly higher decay of phenylalanine and phenyl-*d*_*5*_-alanine was observed under oxic incubations (100% decay in 3 days) compared with ca. 16% remaining after 3 days under anoxic incubations (Wilcoxon test p < 0.05, Fig. 3). Decay of phenylalanine and phenyl-*d*_*5*_-alanine was not observed in abiotic controls nor in blank incubations (Supplementary Fig. 1B).

### Experimental microbial decay of toluene

In the presence of either NO_3_^−^ or SO_4_^2−^, about 80% of the deuterated and non-deuterated toluene produced by day three had disappeared within 1 day (Fig. 3A,B), whereas no decay was detected in the absence of electron acceptors, where toluene and toluene-*d*_*5*_ remained at ca. 80 µM until the end of the experiment (Fig. 3C, Wilcoxon < 0.05). Evidence of sulfate reduction through production of HS^−^ during the incubation with SO_4_^2−^ is provided by the mass spectrum (Supplementary Fig. 2).

### Year-round and depth variability of toluene in the water column

**Figure 1:**
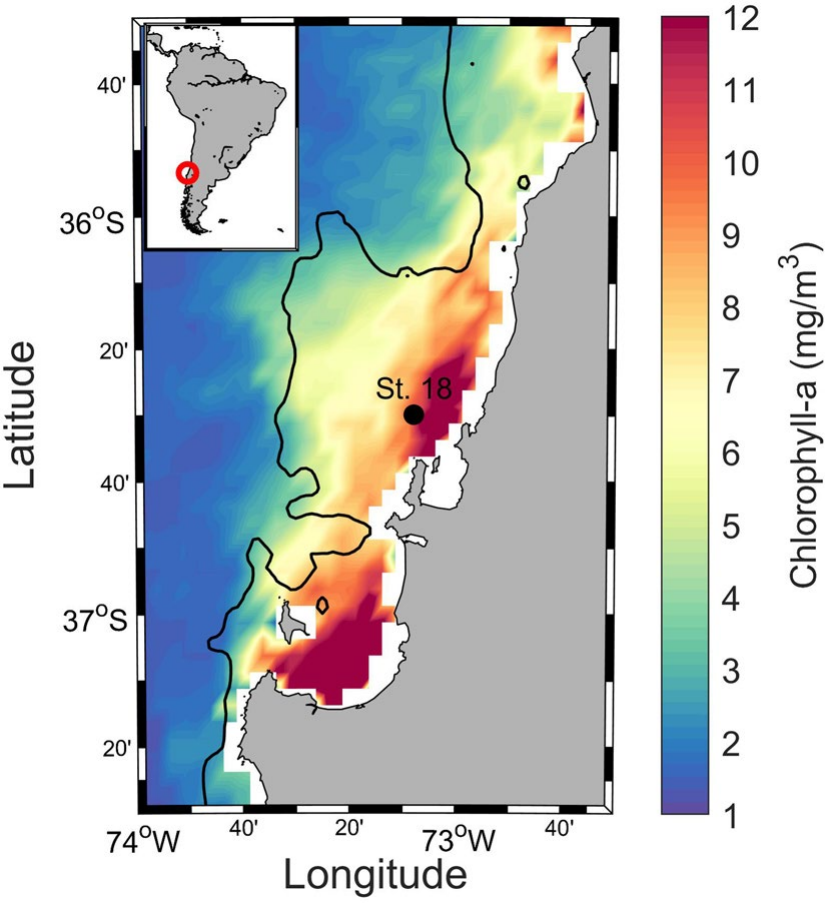
Geographical location of the study site at Station 18, off Concepción in central Chile. Color scale represents the average chlorophyll-a concentration observed between November 2009 and January 2011. The black solid line shows the 200 m isobath. The map was generated using Ocean Data View^40^).

The water column of the study area (Station 18, 90 m depth, Fig. 1) exhibits a distinct annual cycle where suboxic conditions (lower than 22 µM O_2_) below 40 m depth are observed for most of the year. However, during austral winter, O_2_ concentrations increase throughout the water column (Fig. 4A), as a result of the intrusion of the well-oxygenated and less saline ESPTW (Fig. 4B) on the continental shelf off Concepción. During austral summer, salinity increases due to evaporation and entrainment from below, producing summer ESPTW (Fig. 4B). This pattern is consistent with previous investigations on the dynamics of the OMZ off Concepción^8,34–39^.

Toluene concentrations varied between 5 and 96 nM, being lower throughout the whole water column during austral autumn–winter (< 20 nM), but much higher in deeper waters (> 40 m) during austral spring–summer between November 2009, January–March and September–December 2010, and January 2011 (Fig. 5A), consistent with a stratified distribution linked to water mass dynamics (Fig. 5B). Thus, lower concentrations of toluene tend to be associated more with ESPTW, whereas higher concentrations are observed in ESSW (Fig. 5B).

## Discussion

**Figure 2:**
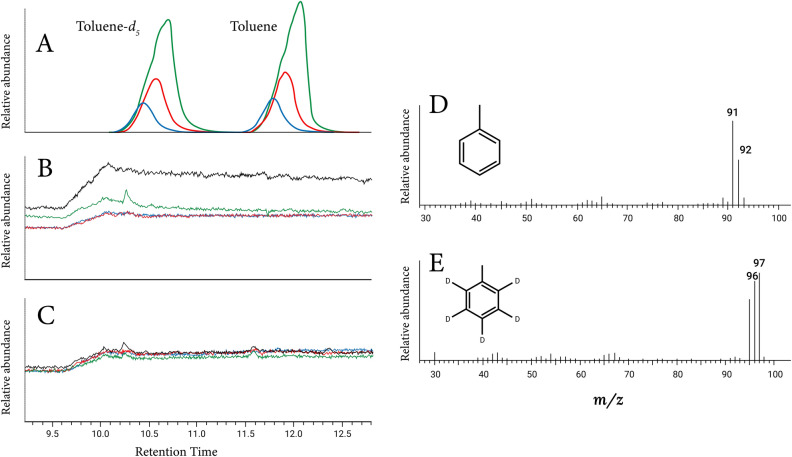
(**A**) Overlaid gas chromatograms showing retention times of toluene and toluene-*d*_*5*_ in incubations anoxic-1 (green line), anoxic-2 (red line), and anoxic-3 (blue line). (**B** and **C**) Overlaid chromatograms from control incubations of synthetic seawater (black line) and treatments without inocula (green, red and blue lines, as in caption A) amended with phenylalanine (**B**) and phenyl-*d*_*5*_*-*alanine (**C**). (**D**) Mass spectrum of toluene (retention time 11.9 min) detected in both environmental samples and in anoxic incubations with phenylalanine as substrate. (**E**) Mass spectrum of deuterated toluene (retention time 10.2 min) produced in anoxic incubations with l-phenyl-*d*5-alanine.

**Figure 3:**
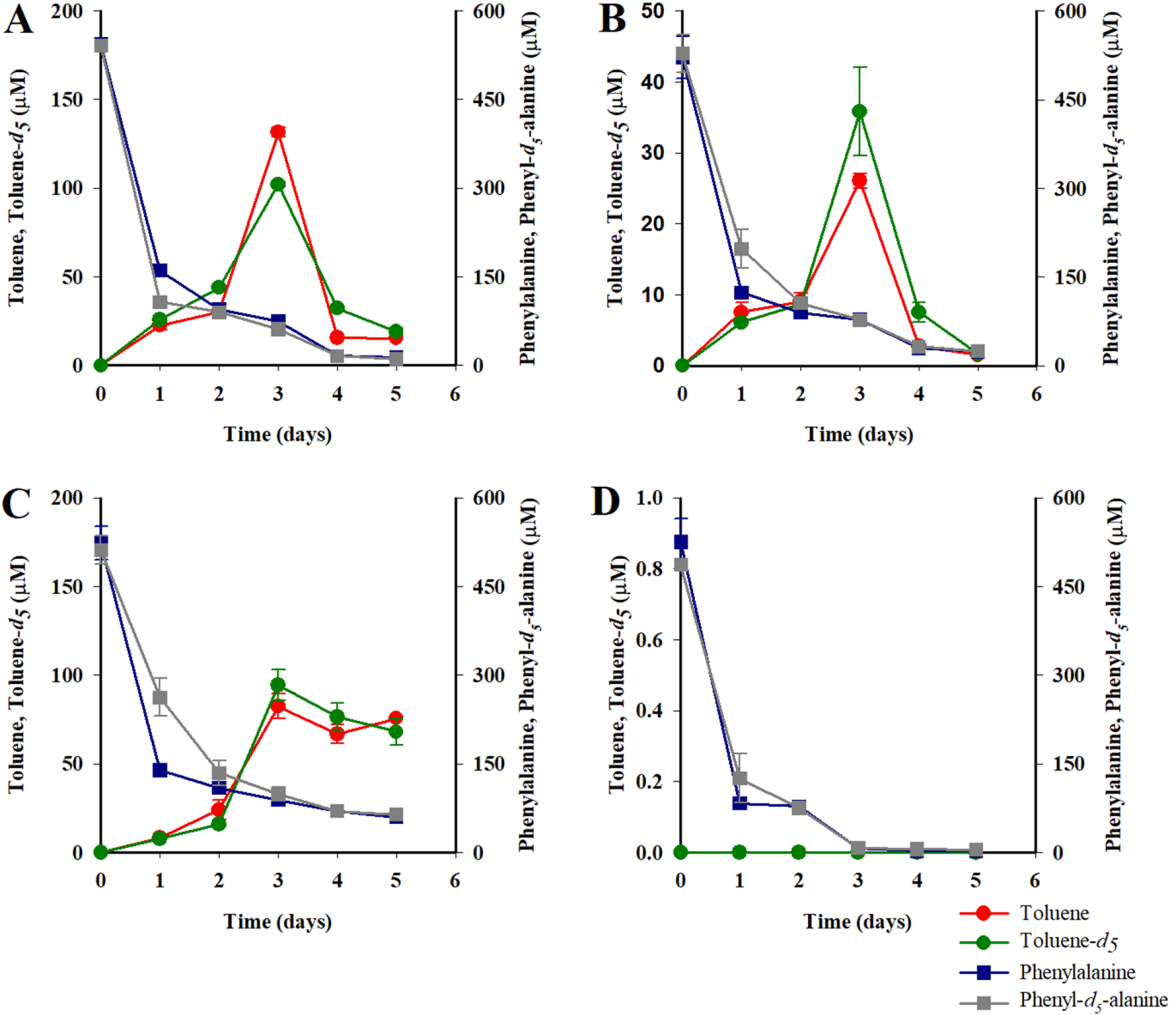
Time course of production of toluene and toluene-*d*_*5*,_ and decay of phenylalanine and phenyl-*d*_*5*_-alanine in (**A**) anoxic-1 incubations (with excess nitrate), (**B**) anoxic-2 incubations (with excess sulfate), (**C**) anoxic-3 incubations (without electron acceptor added), and (**D**) oxic incubations.

Our incubation experiments clearly demonstrated that the microbial assemblage from OMZ waters synthesizes toluene from phenylalanine as an organic substrate under anaerobic conditions (Figs. 2, 3). This corroborates previous observations in bacterial cultures of *Clostridium*^12^ and *Tolumonas*^16^, in anoxic lake waters^41^ and sediments^15^, during anaerobic sludge digestion^13,14^, and phytoplankton cultures^18^. Furthermore, the biosynthesis of toluene-*d*_*5*_ from the deuterated aromatic ring of phenyl-*d*_*5*_-alanine shows that phenylalanine was indeed the precursor of toluene (Fig. 2A,D,E). The production of labeled toluene from the aromatic ring of the deuterated phenylalanine suggests a glycyl radical toluene synthase attacks on phenylalanine or phenylacetate methylene β-carbon^42^. Glycyl radical enzymes are proteins that belong to the enzymes of the Fe–S cluster, one of the oldest proteins of the planet^43,44^. The gene encoding for Fe–S proteins assembly was recently identified in the metagenome of Marinimicrobia bacteria (MAG-SAR406), a pervasive OMZ bacterium, detected in the Oxygen Minimum Zone of the Tropical Pacific Ocean^45–47^, with the capability for hydrogen and carbon monoxide oxidation, arsenic metabolism, cellulose degradation, and partial denitrification. Being a permanent feature of the modern ocean, we hypothesize that these radical enzymes are widely distributed in the microbial assemblage inhabiting the OMZ. Additionally, bacteria from the Acidobacteria phylum have been detected in OMZ's waters of northern Chile^48^, and the toluene-producing enzyme phenylacetate decarboxylase (PhdB) and its cognate, a radical S-adenosylmethionine activating enzyme (PhdA), and the genes encoding for the enzyme were identified from the metaproteome and metagenome of an Acidobacteria isolated from an anaerobic sewage sludge^9^. Acidobacteria were found exclusively in OMZ waters compared to surface and deeper water masses in northern Chile and are therefore a potential producer of toluene; however, their abundance is lower than other pervasive bacterial groups present in these waters^48^.

Reactions catalyzed by phenylacetate decarboxylases are endothermic; therefore, microbial production of toluene has been associated with gaining selective advantages other than energy conservation^9^. Toluene-producing bacteria of the OMZ could produce toluene as a mechanism of negative allelopathy against nanoheterotrophs predators abundant in OMZ waters^49,50^.

Toluene could also be synthesized for intracellular pH homeostasis regulation since phenylacetate decarboxylation removes protons from the cytoplasm, thus promoting cellular alkalinization^9^. This mechanism could represent a selective advantage for toluene-synthesizing OMZ microorganisms to thrive in the moderately acidic conditions of OMZ waters (pH 7.8) due to the intrusion of the more acidic ESSW^51–53^, and the co-occurrence of metabolic protongenic reactions during glucose, acetate, and Stickland fermentation^8^. In addition, depletion of protons in the cytoplasm allows the development of proton-motive force that will generate metabolic energy for toluene-producers microbes^54^.

Although the experiment with deuterated phenylalanine unequivocally confirmed phenylalanine as a precursor for toluene biosynthesis, an expected 1:1 stoichiometric production of toluene from phenylalanine^10^ was not demonstrated. The observed toluene production accounted for only 5–21% of phenylalanine decay under all anoxic treatments (Fig. 3A–C). Phenylalanine can undergo oxidation to CO_2_^55^, thus partially explaining the relatively low toluene yield. Anaerobic catabolism of phenylalanine without production of toluene has been shown to produce phenylacetate, phenylpropionate, and phenylacetate in fermenting bacteria *Clostridium,* and producing phenylacetate in denitrifier *Thauera aromatic*^11,56,57^. Thus, decay of phenylalanine in our incubations could result from the activity of a toluene-producing glycyl radical enzyme and from anaerobic catabolism mediated by benzoyl-coenzyme A^11,56–58^, with phenylacetate as the intermediate substrate in both pathways. Another factor that could influence the 1:1 stoichiometry in our incubations is the concurrent microbial oxidation of toluene as it is being produced, as already shown for other environments^59–66^. In consequence, observed changes in toluene concentrations during incubations will correspond to net production.

Toluene microbial decay by anaerobic oxidation coupled to denitrification and partial denitrification are the most thermodynamically favorable reactions (ΔG > 2500 kJ mol^−1^) compared with toluene oxidation with NO_2_^−^ and SO_4_^2−^ and fermentation (Table 1). Metagenome-assembled genomes from the eastern tropical Pacific OMZ contain genes involved in partial denitrification^67^, supporting the feasibility of this metabolism in OMZ waters.

**Table 1:** Anaerobic toluene degradation reactions in laboratory incubations of suboxic waters collected in January 18, 2011 at Station 18 off Concepción at 65 m depth in the OMZ.

Metabolic reaction	Chemical equation	ΔG^0^(kJ/mol toluene)	ΔG (kJ/mol toluene) OMZ water conditions	ΔG (kJ/mol toluene) laboratory conditions
Toluene oxidation coupled to denitrification	C_7_H_8_ + 7.2NO_3_^−^ + 0.2H^+^ → 3.6N_2_ + 7HCO_3_^−^ + 0.6H_2_O	− 3395	− 3454	− 3530
Toluene oxidation coupled to partial denitrification	C_7_H_8_ + 18NO_3_^−^ + 3H_2_O → 18NO_2_^−^ + 7HCO_3_^−^ + 7H^+^	− 2105	− 2585	− 2972
Toluene oxidation coupled to nitrite reduction	C_7_H_8_ + 12NO_2_^−^ + 5H^+^ → 6N_2_ + 7HCO_3_^−^ + 3H_2_O	− 408	− 337	− 392
Toluene oxidation coupled to sulfate reduction	C_7_H_8_ + 4.5SO_4_^2−^ + 3H_2_O → 2.25H_2_S + 2.25HS^−^ + 7HCO_3_^−^ + 0.25H +	− 218	− 381	− 350
Toluene fermentation	C_7_H8 + 21H_2_O → 18H_2_ + 7HCO_3_^−^ + 7H^+^	+ 759	+ 293	+ 399

Gibbs energy (ΔG) was calculated assuming substrate and product activities, temperature, and pH of the suboxic water column conditions for austral spring at the sampling site as well as for the conditions of incubations. For comparison, Standard Gibbs energy (ΔG^0^) values are shown.

In anaerobic toluene-degrading communities in aquifer sediments, variability in NO_2_^−^ and NO_3_^−^ concentrations determines the fate of reduction of NO_3_^−^ (NO_2_^−^ or N_2_) by modulating changes in the microbial community of denitrifiers that totally or partially denitrifies^67^. In our study site, the distribution and availability of NO_2_^−^ showed a clear vertical and temporal pattern with the highest concentrations during austral spring and summer and restricted to the deepest layers, that resembles the vertical distribution of toluene concentrations throughout a year cycle (Supplementary Fig. 3A,C), both significantly correlated (Supplementary Table 1), suggesting the occurrence of partial denitrification. On the contrary, NO_3_^−^ concentration decreases in the deepest layer during austral summer (Supplementary Fig. 3B), and the year-round pattern resembles the upwelling and productivity cycles in the study area, as shown by negative correlations (Supplementary Table 1) with temperature (an indicator of upwelling) and fluorescence (an indicator of phytoplankton productivity). Taken together, it suggests that partial denitrification during degradation of toluene could be occurring in these waters, apparently associated with the year cycle in nutrient availability, thus providing NO_2_^−^ to anammox bacteria in these waters^68^, unveiling a novel and hitherto unknown connection of C and N cycling in OMZ waters.

The anoxic incubations also revealed that ~ 80% of produced toluene disappeared within 1 day in the presence of NO_3_^−^ or SO_4_^2−^, whereas in the absence of these electron acceptors, toluene remained at ~ 80 ± 4 µM on day 3 and decayed slightly to less than 75 ± 6 µM by day 5 (Fig. 3C). Thermodynamic considerations support this since fermentation of toluene is endergonic under both experimental and field conditions (Table 1) due to the production of H_2_ during its disproportionation that drives ΔG to be > 0. However, partial pressure of H_2_ can be reduced by an electron-acceptor microorganism in a microbial syntrophic consortium, thus rendering an overall exothermic fermentation reaction, as shown in laboratory co-cultures^66^. Moreover, in a syntrophic coculture, Meckenstock et al.^69^ also detected that syntrophic degradation is 2–3 times slower than toluene oxidation by sulfate. Our relatively short experimental incubations preclude conclusions regarding whether toluene would remain at 80 ± 4 μM (Fig. 3C) or would slowly decay in the absence of external electron acceptors, given sufficient microbial consortium growth.

**Figure 4:**
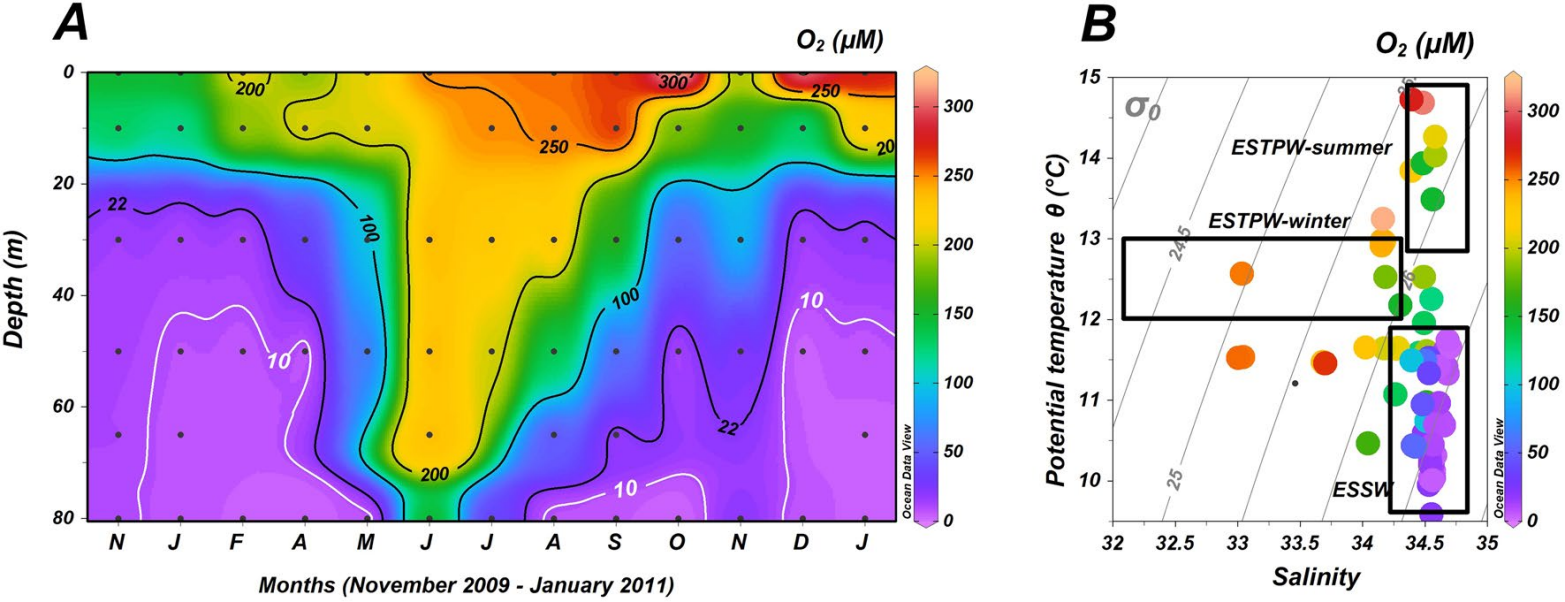
(**A**) Dissolved oxygen time-series data from November 2009 throughout January 2011. The white line represents the 10 µM O_2_ isoline. Black dots represent sampling points. (**B**) Potential temperature (ϴ)/salinity diagram from depth profiles taken at Station 18, off Concepción. Data points are color-coded (side color bar) for oxygen concentrations. Rectangles indicate temperature and salinity ranges for Equatorial Subsurface Water (ESSW) and Eastern South Pacific Transition Water (ESPTW) in austral winter and summer. Grey dotted lines represent isopycnals as sigma-t (σ_0_).

**Figure 5:**
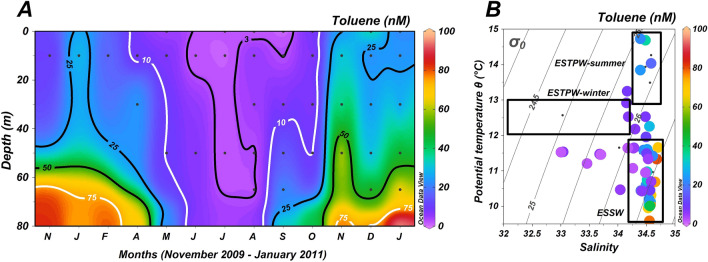
(**A**) Toluene time-series data from November 2009 to January 2011. White lines represent the 10 and 75 µM toluene isolines. Black dots represent sampling points. (**B**) Potential temperature (ϴ)/salinity diagram from depth profiles taken at Station 18, off Concepción. Data points are color-coded (side color bar) for toluene concentrations. Rectangles indicate temperature and salinity ranges for Equatorial Subsurface Water (ESSW) and Eastern South Pacific Transition Water (ESPTW) in austral winter and summer. Grey dotted lines represent isopycnals as sigma-t (σ_0_).

Our study site (Station 18) is located 18 nautical miles from the coast of Concepción, a major city of Chile, and therefore input from the urban, maritime, port and industrial activities is conceivable since toluene is universally used in industry (principally as a solvent and gasoline additive) and in domestic products^70^. However, depth profiles of toluene in the water column (Fig. 5A and Supplementary Fig. 4) indicate natural biogeochemical processes. In austral spring, summer, and autumn, increased concentrations with depth are consistent with a deeper source for toluene in O_2_-depleted waters (Figs. 4A and 5A). Despite our evidence for potential toluene production in the water column, diffusion from sediments is an additional source that cannot be ruled out, as shown in other coastal anoxic sediments^70,71^.

Circulation of water masses forced by atmospheric conditions represents a fundamental control of the annual cycle at the study site. Oceanographic Station 18 (Fig. 1) is at the southernmost limit of the extensive OMZ of the Humboldt Current System^72^. It is subject to alternate intrusions of oxygenated subantarctic waters during austral autumn–winter and of anoxic and hypoxic waters associated with Equatorial Subsurface Waters (ESSW)^37^. Concentrations of toluene exceeding 50 nM occurred principally below the 10 µM O_2_ isoline (Fig. 4A), conditions favorable for the activity of the glycyl radical decarboxylase enzyme^9,10^, otherwise irreversibly inactivated by O_2_^10,19,21^.

The vertical distribution of toluene in the water column appears to be inversely related to dissolved oxygen at the study site (Figs. 4A and 5A), with a significant negative correlation between the two (Spearman correlation, ρ = − 0.6, p < 0.05, Supplementary Table 1). Moreover, toluene concentrations correlate negatively with temperature and positively with salinity (Spearman correlation, p < 0.05, Supplementary Table 1), indicative of the annual cycle of upwelling of ESSW and intrusions of ESPTW (Fig. 5B), as previously observed^8,37^. Significant associations of toluene concentrations with NO_2_^−^, PO_4_^3−^, and volatile fatty acids (VFA; Spearman correlations; p < 0.05; Supplementary Table 1) were also observed. These are considered to be molecular indicators of fermentation^8^ and denitrification^6,73^ and provide the chemical environment for toluene production in the water column.

Biosynthesis of toluene from deuterated and non-deuterated phenylalanine was not detected in oxic incubations (Fig. 3D), as might be expected^10^. This corroborates our observations that the lowest concentrations of toluene occurred in well-oxygenated and low salinity subantarctic waters that form ESPTW in winter and summer (Fig. 5B). Because subantarctic waters are well oxygenated, we would anticipate that no production of toluene occurs in these waters, and we suggest that the relatively small inventory of toluene in ESPTW results from mixing with OMZ waters (Fig. 5). We cannot rule out that OMZ’s pervasive bacterial groups SAR202 and SAR406 may degrade aromatics and high molecular weight recalcitrant dissolved organic matter^47,74^ using monooxygenase enzymes when oxygenated waters intrude the continental shelf. Therefore, the lower concentrations of toluene observed in the most oxygenated waters of the study site (e.g., ESPTW), could be the net result of input from OMZ waters and microbial aerobic degradation as O_2_ availability increases during mixing (Fig. 4A,B).

The average depth-integrated toluene inventory of 3 mmol toluene m^−2^ (Supplementary Fig. 3) represents an estimated reservoir of about 2 million tons of toluene for the whole OMZ off Chile of ca. 10 million km^2^^72^, is about 50% higher than the inventory of CH_4_ and near 20 times lower than that of VFA (Supplementary Table 2), putative representative molecules of anaerobic metabolism. The estimated inventory of toluene is a significant quantity compared with the annual toluene production of the European Union of 1–10 million tons^71^.

## Conclusions

In laboratory incubations of OMZ waters off central Chile, microbial assemblages anaerobically biosynthesize toluene from phenylalanine, a finding consistent with the year-round pattern of toluene concentrations in the water column in the upwelling ecosystem of the Humboldt Current System. A fraction of this toluene is exported to surrounding oxygenated waters, namely the subantarctic Eastern South Pacific Transition Water (ESPTW).

Microbial production of toluene increases cellular pH, perhaps counteracting the external acidic media of the OMZ (pH 7.8). The field and laboratory data analysis suggest that, as shown for toluene-degrading communities in aquifer sediments, the availability of NO_2_^−^ to oxidize toluene could determine the fate of the reduction of NO_3_^−^ (NO_2_^−^ or N_2_).

Our estimate of the potential reservoir of toluene in the OMZ off Chile is in the order of 2 million tons, within the range of CH_4_ and VFA inventories, putative representative molecules of anaerobic metabolism, as well as in the range of annual industrial production of toluene for the European Union. Warming and eutrophication of coastal waters are likely to further promote hypoxic conditions, enhance anaerobic metabolism, and potentially reveal reservoirs of various organics produced by the OMZ microbiome.

## Methods

### Sampling

#### Water samples

Water samples were collected onboard the research vessel Kay-Kay II (Universidad de Concepción) quasi-monthly between November 2009 and January 2011 (13 sampling dates). Seawater was collected at depths of 0, 10, 30, 65, 70, and 80-m using a rosette system equipped with 10-L Niskin bottles. Subsamples of 50 mL volume were transferred onboard to pre-combusted (450 °C, 4 h) gas tight borosilicate Wheaton bottles (darkened with aluminum foil) under a N_2_ atmosphere—using glove bags (Aldrich AtmosBag) to avoid O_2_ contamination—then immediately poisoned with HgCl_2_ (0.001%) to arrest microbial activity. Bottles were crimp-sealed with ultra-pure bromobutyl stoppers (Wheaton) and stored in the dark at 4 °C until further analysis of toluene in the laboratory (within 12 h). Data for temperature, salinity, O_2_, NO_3_^−^, NO_2_^−^, PO_4_^3−^, NH_4_^+^ and fluorescence were provided by the COPAS Center *Time Series Oceanographic Station 18* (FONDAP COPAS) and the program *Microbial Initiative in Low Oxygen off Concepción and Oregon* (Moore Foundation http://mi_loco.coas.oregonstate.edu). Volatile fatty acid (VFA) data were taken from Srain et al.^8^.

### Inocula and incubation media

Inocula of seawater for laboratory experiments were obtained on January 18, 2011, from 65 m depth (10.3 °C, 34.6 PSU, 4.0 µM O_2_) and transferred to 10 mL serum vacuum-tubes BD. Tubes were maintained at 10 °C in darkness until arrival at the laboratory. Inocula and reagents were manipulated in a laminar flow hood LABCONCO Class II Type IIA under a N_2_-saturated atmosphere (glove bags Aldrich Atmosbag) achieved by bubbling with N_2_ (99.9% purity). O_2_-sensitive methylene blue (Resazurin 0.0001%, Wolfe^75^; McDonald et al.^76^) was added to anoxic incubation vessels to detect traces of O_2_ contamination (> 0.7 µM). Artificial seawater for anoxic incubations was prepared according to Lovley^77^.

### Experimental setup

Experimental incubations were conducted in duplicate by transferring 30 mL of artificial seawater into darkened pre-combusted (450 °C, 4 h) borosilicate glass bottles (60 mL, Wheaton), under a N_2_ saturated atmosphere. l-Phenylalanine in excess (500 µM, CAS 63–91-2) and d-glucose (10 µM, CAS 50-99-7), were added as carbon and nitrogen sources, and inoculated with 10% v/v of seawater collected from the OMZ. Bottles were sealed with ultra-pure bromobutyl stoppers, keeping a headspace volume of 20 mL for sampling of volatile compounds. Incubation of bottles was carried out at 10 °C in darkness, with constant orbital agitation (180 rpm). Incubations were conducted for 5 days with the headspace sampled (500 µL with a Hamilton gas-tight syringe 1750) every ca. 24 h.

Experimental treatments were (in parenthesis the initial final concentration added).

1. Anoxic-1 incubation under denitrifying conditions: Phenylalanine (500 µM), glucose (10 µM) and NO_3_^−^ (800 µM). This same concentration of NO_3_^−^ was subsequently added daily under a N_2_-saturated atmosphere using a microliter syringe Hamilton (500 µL) previously flushed with N_2_.
2. Anoxic-2 incubation under sulfate reduction conditions: Phenylalanine (500 µM), glucose (800 µM) and SO_4_^2−^ (450 µM), amended with 5% v/v of reducing solution (Na_2_O_2_S × 5H_2_O and cysteine) to promote sulfate reduction. This same concentration of SO_4_^2−^ was subsequently added daily under N_2_-saturated atmosphere using a microliter syringe Hamilton (500 µL) previously flushed with N_2_.
3. Anoxic-3 incubation under fermentative conditions: Phenylalanine (500 µM) and glucose (800 µM) without external electron acceptors.
4. Oxic incubation: Phenylalanine (500 µM), glucose (800 µM) and O_2_ (370 µM). Incubation flasks were oxygenated to saturation (8.9 mL L^−1^ at 10 °C and 0.21 atm, Henry’s Law) with synthetic air (*ca*. 20% O_2_, 80% N_2_, 99.99% purity). Five mL aliquots of pure synthetic air were added daily to the incubation flasks using a 1000 µL gas-tight syringe Hamilton.
5. Experiments Anoxic-1, Anoxic-2, Anoxic-3, and Oxic were also repeated using deuterated phenylalanine (500 µM l-phenyl-*d*_*5*_-alanine, CAS 284664-89-7), instead of phenylalanine, to confirm that toluene synthesis came from phenylalanine.

Anoxic conditions were achieved by gently bubbling N_2_ into incubation bottles for 15 min to displace traces of O_2_. Abiotic controls with substrates were incubated without OMZ inocula for each treatment.

### Analysis of toluene in ambient and experimental samples

Analysis of toluene was carried out through Headspace Solid Phase Micro–Extraction coupled to Gas Chromatography with MSD detection (HS–SPME–GCMS). Twenty-five mL of water was removed from each sample (n = 3), under a N_2_ saturated atmosphere, to generate a headspace. Environmental and experimental samples were placed on a thermostatic hotplate at 20 °C, with constant stirring (using an acid-cleaned and sterilized stirring bar) for 5 min to reach partition equilibrium between aqueous and gas phases. Polar and non-polar volatile organic compounds were adsorbed using a fiber of 85 μm Carboxen/PDMS Stable Flex (SUPELCO). The fiber was inserted into the insertion septum of the gas bottle and exposed to the gas phase (headspace). After adsorption of analytes, the fiber was placed in the injection port of the gas chromatograph and exposed for 5 min at 250 °C for desorption of the collected gaseous analytes. GC–MS analyses were conducted using a gas chromatograph Agilent 6890 N series coupled to a mass spectrometer Agilent 5973 Network. The mass spectrometer was operated in electron impact mode (70 eV) and spectra of toluene standards and incubation samples were acquired in full scan mode (m/z 40–600, 2.6 s^−1^), with spectra for environmental samples acquired using selective ion monitoring (SIM): toluene (tropylium ion m/z 91). Chromatographic separation was achieved using a HP–Plot/Q column 30 m (0.32 mm diameter, 0.20 μm film thickness), using He as carrier gas. The oven temperature program started at 100 °C (5 min), ramped to 230 °C at 50 °C min^−1^ (held 2 min), to 250 °C at 50 °C min^−1^ (held 7 min).

Quantification of toluene and deuterated toluene (toluene-*d*_*5*_) in both scan and selective ion monitoring (SIM) modes was determined using calibration curves (R^2^ > 0.994) for which toluene standards (HPLC grade, ≥ 98.8% purity, Fisher) were added to artificial seawater in the range 1 nmol L^–1^ –1 mmol L^−1^. Concentrations of toluene in the gas phase were calculated as C_HS_ = C_ap_/(K + (V_HS_/V_S_)) with the partition coefficient K = C_AP_/C_HS_. C_HS_ is the concentration in the headspace, C_AP_ is the concentration in the aqueous phase, and V_HS_ and V_S_ are headspace and sample volumes^78^, resulting in a partition coefficient of 0.9. Detection limits were calculated from the slopes and residual standard deviations derived from linear regressions of calibration curves (3 times residual error times slope)^79^. Limit of detection for toluene was 1 nmol L^−1^, whilst limit of quantification was 5 nmol L^−1^. Synthetic seawater was analyzed for toluene.

### Analyses of dissolved free amino acids (DFAA)

Concentrations of DFAA from incubations, and from Station 18, were quantified as OPA-derivatized adducts^80^ with a Shimadzu LC-10AT HPLC coupled to a Shimadzu RF-10Axl fluorescence detector (set at excitation/emission of 340/450 nm), column oven CTO 10As and autosampler (Shimadzu SIL 10 ADvp). Aliquots of 0.6 mL, mixed with 0.4 mL methanol, were derivatized in the autosampler with 60 μL ortho-phthalaldehyde/2-mercaptoethanol reagent and 100 μL sodium acetate buffer 0.1 N, pH 5, and then injected (50 μL) into the HPLC. Fifteen amino acids (asp, glu, ser, his, gly, thr, arg, ala, tyr, val, met, phe, ile, leu, lys) were separated using an Alltima C18 (5 mm, 250 × 4.6 mm) column maintained at 40 °C, with a mobile phase of 5% tetrahydrofuran in 25 mM sodium acetate and methanol, and at a flow rate of 1 mL min^−1^. A gradient of 25–30% methanol in 35 min, 30–50% in 7 min, 50–60% in 18 min, 60–100% in 12 min was used. Initial conditions were restored within 7 min, and the column equilibrated for 10 min between injections. Amino acids were identified and quantified by comparison with chromatograms of a standard amino acid mix (Pierce 20088) run under the same conditions every 10 injections. The coefficient of variation for quantification of duplicate samples was 9.4%.

### Statistical analysis

Since homogeneity of variances (Levene test) and normality of variables (Shapiro–Wilk test) were not fulfilled, we tested for significant differences between environmental data using the non-parametric Kruskal–Wallis ANOVA test. Statistical differences between experimental treatments were determined by using the Wilcoxon matched pairs test. Correlations were examined using Spearman R coefficients.

### Thermodynamic calculations

Calculated Gibbs Energy values (ΔG) for proposed oxidation reactions of toluene in the suboxic water column were calculated using activities of substrates and products, temperature and pH of the water depth of inoculum collection in austral spring or from literature. The following data and sources were used: temperature (10.2 °C), NO_2_^−^ (5 µM) and NO_3_^−^ (25 µM) from the COPAS Center Oceanographic Time Series database (W. Schneider, curator), pH (7.8, this study), toluene (0.09 µM, this study), SO_4_^2−^ (28 mM)^81^, HCO_3_^−^ (2 mM)^81^, N_2_ (389 µM)^82^, HS^−^ (1 µM)^83,84^. Activities of N_2_, NO_2_^−^, NO_3_^−^, SO_4_^2−^ and HS^−^ in experimental incubations were determined stoichiometrically from the chemical equations of the proposed reactions. Thermodynamic calculations were carried out using THERMODYN software^85^.

## Supplementary Information

Supplementary Information 1.

Supplementary Information 2.

Supplementary Information 3.

## Data Availability

All data generated or analysed during this study are included in this published article and its supplementary information files.
