# Supplementary material for: Microbial production of toluene in oxygen minimum zone waters in the Humboldt Current System off Chile
Source: Sci Rep. 2022 Jun 23;12:10669. doi: 10.1038/s41598-022-14103-2 (PMC9226047; doi:10.1038/s41598-022-14103-2)

**Microbial production of toluene in oxygen minimum zone waters in the Humboldt Current System off Chile**

Benjamín M. Srain^1,&^*, Silvio Pantoja-Gutiérrez^1,2^*

^1^Departamento de Oceanografía and Centro de Investigación Oceanográfica COPAS Sur-Austral, Universidad de Concepción, Concepción, Chile

^2^Centro de Investigación Oceanográfica COPAS Coastal, Universidad de Concepción, Concepción, Chile

^&^ Present address Programa de Estudios Ecosistémicos del Golfo de Arauco (PREGA). Universidad de Concepción, Concepción, Chile

* Corresponding authors

*Email addresses:* [*bsrain@udec.cl*](mailto:bsrain@udec.cl)*,* [*spantoja@udec.cl*](mailto:spantoja@udec.cl)

**Supplementary table 1.** Combined Spearman correlation matrix for chemical and physical variables measured at Station 18. Significant correlations are highlighted in bold

**Supplementary table 2**: Inventories of volatile organic compounds in the coastal upwelling period (2010-2011).

|  |  |  |  |  |
| --- | --- | --- | --- | --- |
|  | 2010 |  |  | 2011 |
| Inventory (µmol m^-2^) | October | November | December | January |
|  |  |  |  |  |
| Toluene (this study) | 1020 | 2717 | 2597 | 3750 |
| Volatile fatty acids ^1^ | 28000 | 35000 | 96000 | 82000 |
| Methane ^2^ | 1080 | 1952 | 1811 | 2611 |

1. Acetate, 66%, isobutyrate, 33% and isovalerate, 1%. Srain, B. M. *et al.* Fermentation and Anaerobic Oxidation of Organic Carbon in the Oxygen Minimum Zone of the Upwelling Ecosystem Off Concepción, in Central Chile. *Front. Mar. Sci.* (2020) doi:10.3389/fmars.2020.00533.

2. Florez-Leiva, L., Damm, E. & Farías, L. Methane production induced by dimethylsulfide in surface water of an upwelling ecosystem. *Prog. Oceanogr.* (2013) doi:10.1016/j.pocean.2013.03.005.

**Supplementary figure captions**

**Supplementary figure 1.** Temporal changes in concentrations of: (A) toluene and toluene-*d_5_* and (B) phenylalanine and phenyl-*d_5_*-alanine in control abiotic incubations. Control 1 (anoxic-1 amended with phenylalanine); control-2 (anoxic-1 amended with phenyl-*d_5_*-alanine); control 3 (anoxic-2 amended with phenylalanine); control 4 (anoxic-2 amended with phenyl-*d_5_*-alanine); control 5 (anoxic-3 amended with phenylalanine); control 6 (anoxic-3 amended with phenyl-*d_5_*-alanine); control 7 (oxic amended with phenylalanine); control 8 (oxic amended with phenyl-*d_5_*-alanine).

**Supplementary figure 2.** Gas chromatogram and mass spectrum of hydrogen sulfide from the headspace of incubation bottle at t5 (5 days, Figure 3B) with SO_4_^2-^ as terminal electron acceptor.

**Supplementary figure 3.** Concentrations of (A) nitrite, (B) nitrate, and (C) toluene at Station 18 from November 2009 throughout January 2011.

**Supplementary figure 4.** Toluene depth profiles during the sampling period at station 18 during austral spring-summer (A) and winter (B). Trapezoidal integration of toluene concentration with depth results in an estimated average reservoir of 3 mmol m^-2^.

**Supplementary figure 1.**

**
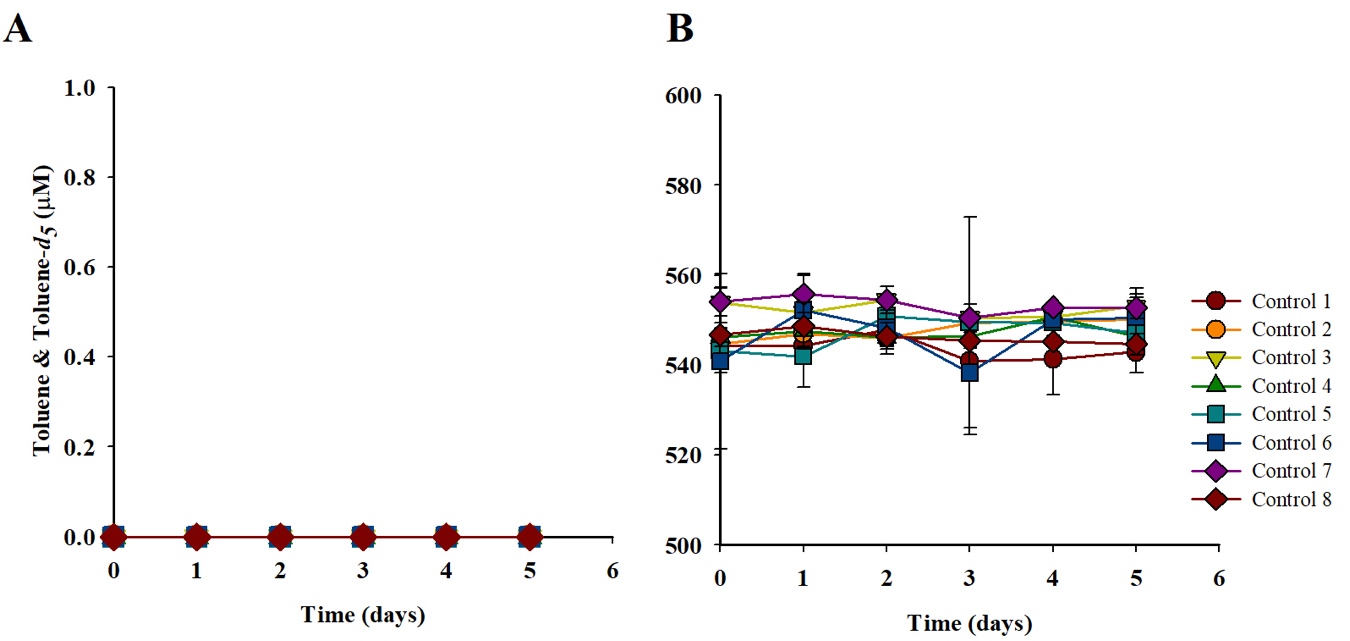
**

**Supplementary figure 2**

**
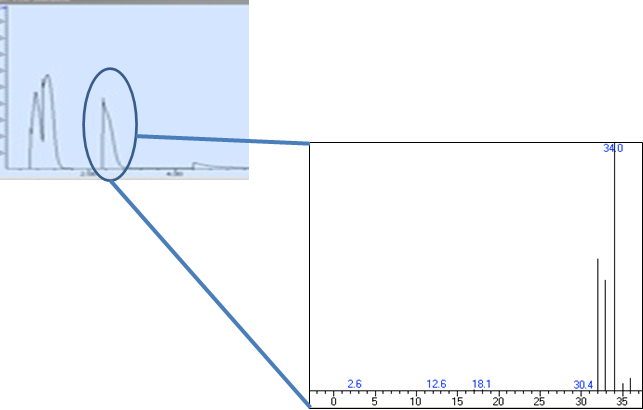
**

**Supplementary figure 3**


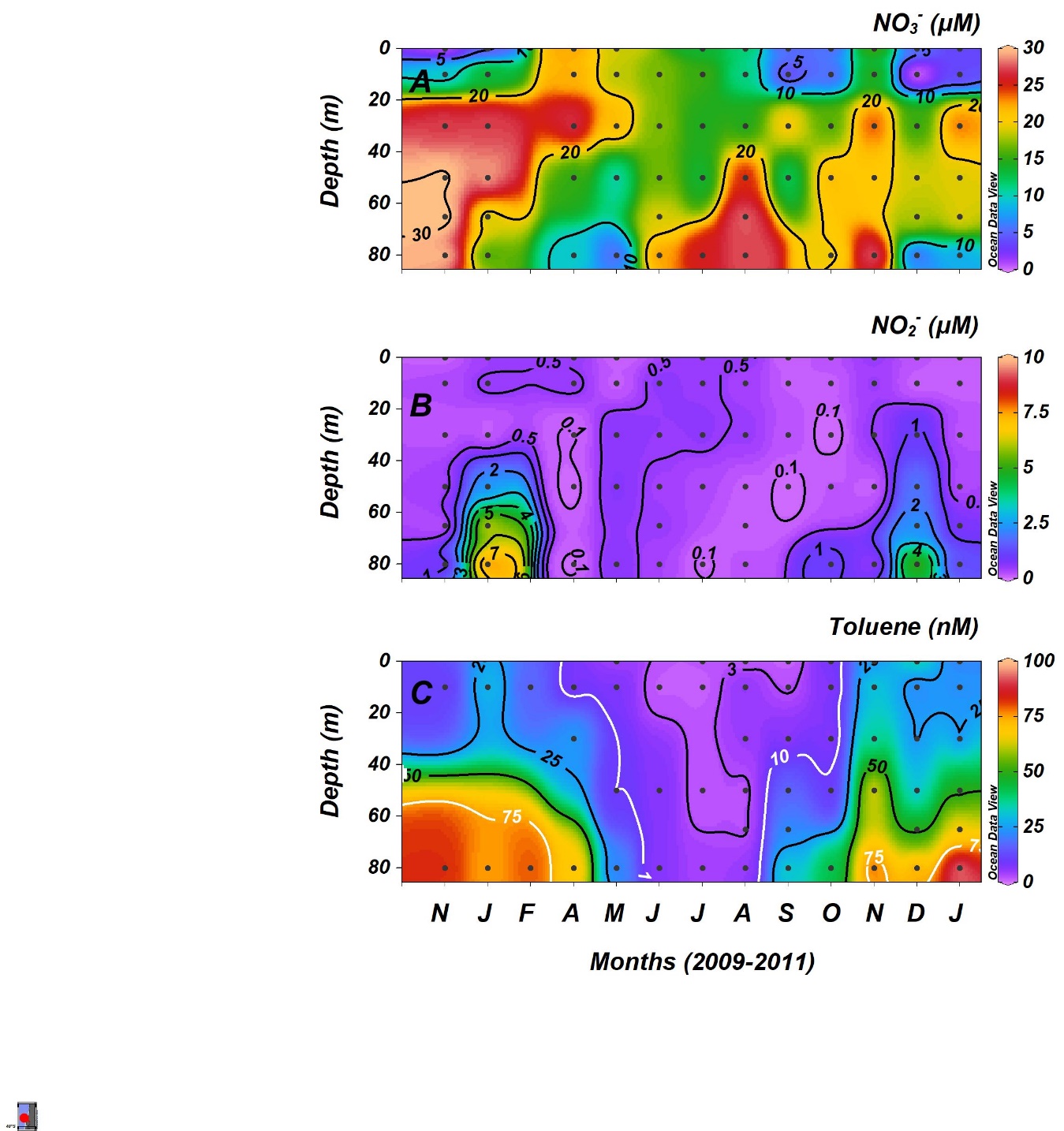


**Supplementary figure 4**


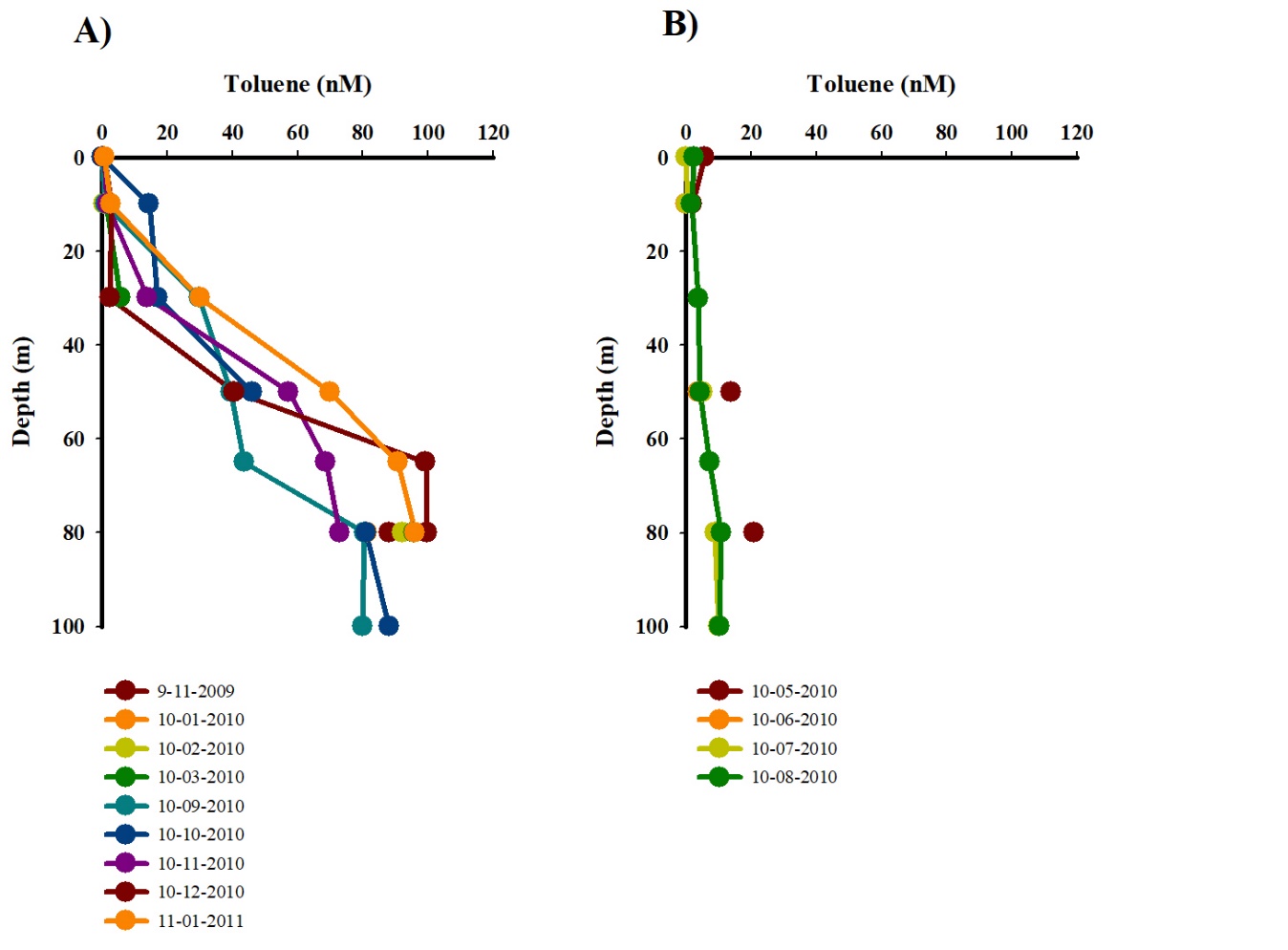

Supplement: Supplementary file 2 — Supplementary Information 2. [file 41598_2022_14103_MOESM2_ESM.docx]
